# Role and mechanism of miR-211 in human cancer

**DOI:** 10.7150/jca.71401

**Published:** 2022-07-18

**Authors:** Lingling Ye, Fen Wang, Jinyan Wang, Hao Wu, Hui Yang, Zhaohui Yang, Haiwei Huang

**Affiliations:** 1 Department of Gynecology, Zhangjiagang First People's Hospital, Zhangjiagang Affiliated Hospital of Soochow University, Zhangjiagang, Jiangsu, China; Department of Oncology, The Affiliated Jiangning Hospital with Nanjing Medical University, Nanjing, Jiangsu, China.; 2 Department of Oncology, The Affiliated Jiangning Hospital with Nanjing Medical University, Nanjing, Jiangsu, China.

## Abstract

MircoRNA (miRNA), which are a group of small, and highly conserved non-coding RNA consisting of 18-25 nucleotides, can modulate gene expression at post-transcriptional level, through complementary binding to the 3ʹ-untranslated region (3ʹ-UTR) of numerous target genes. Emerging evidence indicates that miRNAs play critical roles in tumorigenesis and progression of cancer. Among them, miR-211 has been extensively studied in multiple cancers. The expression of miR-211 significantly varies with cancer types and may be used as a potential prognostic marker for cancer. MiR-211 can regulate multiple biological processes in cancer, including proliferation, apoptosis, metastasis and drug resistance. Additionally, several factors may contribute to the dysregulation of miR-211 in cancer. Consequently, this review aims to discuss the novel findings that highlight latent value of miR-211 in the prognosis assessment and treatment of cancer.

## Introduction

In recent years, research has identified microRNAs (miRNAs) as important endogenous regulators of gene expression in all tissues 1. MiRNAs are small, highly conserved non-coding RNAs which typically consists of 18-25 nucleotides, and they regulate posttranscriptional gene expression. MiRNAs do not entail complete complementarity for target recognition, hence, a miRNA can bind to several target mRNAs by recognizing complementary sites in 3ʹ-untranslated regions (3ʹ-UTR) or in some situations at their 5ʹ-UTR 2, 3. However, the “seed sequence” of the miRNA must perfectly match its target, the “seed sequence,” is the most important sequence for target recognition, is nucleotides 2-8 of the miRNA 1. Dysregulation of one miRNA may regulate various genes expression through binding to numerous sites which have same seed matches 4. By this means, they are involved in various biological processes, for instance, metabolic processes, cell proliferation, apoptosis, metastasis and differentiation 5-7.

A large number of researches identify that miRNAs are related to the pathogenesis of many diseases, particularly cancer 8, 9. Depending on the nature of their target genes, miRNAs can function as tumor suppressors or oncogenes (also known as oncomiR) 10. OncomiRs can inhibit the expression of tumor suppressive target mRNAs, and are frequently upregulated in the disease. Conversely, tumor suppressive miRNAs negatively regulating oncogenic targets, and are downregulated in cancer 11. Furthermore, one miRNA can act as a tumor suppressor in one cancer and as an oncogene in another 12, 13, indicating that conducting research on the same miRNA in different type of cancers is very significant.

Among various miRNAs that have been reported to be dysregulated in cancer, miR-211 has been recognized as one of the miRNAs that play a crucial role in cancer pathogenesis. Regarding its structure, miR-211 is localized in intron 6 of Trpm1 gene at 15q13-q14 14. The miR-211 hairpin is further cleaved into the “guide strand” miR-211-5p and the sister “passenger” strand miR-211-3p. Here, we will describe the different roles, even opposing that miR-211 can play, depending on the tissue context or the cancer type. Most of studies have focused on miR-211-5p and only a few on miR-223-3p; however, in most cases, the strand is not specified. Furthermore, the role of miR-211 in cancer is closely related to its impact on various tumor biological processes, such as cell proliferation 15, apoptosis 16, epithelial-mesenchymal transition (EMT) 17, drug resistance 18, and metastasis 19.

In this article, we will mainly focus on summarizing the mechanisms and functions of miR-211 in tumors and aim to improve our understanding of its significance in the prognosis and treatment of tumors.

## Expression and prognostic value of miR-211 in cancer

### The miR-211 expression in cancer

A great number of researches investigate the expression pattern of miR-211 in the tumor and non-tumor tissues of various cancers (Table 1). The expression of miR-211 is found to be dysregulated in multiple cancers, although upregulation or downregulation is differentially observed, depending on the particular cancer type. In detail, miR-211 is significantly decreased in melanomas compared to melanocytes 20-24. Downregulated miR-211 is observed in papillary thyroid cancer 25, 26. The expression of miR-211 is also greatly downregulated in hepatocellular carcinoma (HCC) 15, 27-30, and gastric cancer 31, 32. Various researches indicate that miR-211 is reduced in renal cell carcinoma (RCC) 33, 34, bladder cancer (BC) 35, ovarian cancer (OC) 36, 37 and cervical cancer 38.

However, the expression of miR-211 is upregulated in osteosarcoma and chondrosarcoma which are tumors Originating in the skeletal system 39, 40. MiR-211 expression is increased in vemurafenib-resistant melanoma cells 41. In oral squamous cell carcinoma (OSCC), enforced miR-211 expression is observed 14. Similarly, Upregulated miR-211 is also observe in colorectal cancer (CRC) 42.

In addition, there is still controversy over the expression of miR-211 in non-small cell lung cancer (NSCLC) 43-45. One study suggests that miR-211 significantly increased in triple-negative breast cancer cells (TNBC) brain metastatic tumors *in vitro* and *in vivo* 46. However, miR-211 is observes decreased in breast cancer in the other two studies 17, 47. Therefore, the expression level of miR-211 varies with cancer type and may be strongly related to tumor heterogeneity and the different stages of tumor progression.

### The prognostic value of miR-211 in cancer

It has been reported that the abnormal expression of mir-211 can be found in a variety of cancers and can be used to predict the prognosis of cancer (Table 1). Higher miR-211 expression is associated with the most advanced nodal metastasis, vascular invasion, and poor prognosis of oral carcinoma 14. In pancreatic ductal adenocarcinoma (PDAC), the result illustrates that low miR-211 is related to higher metastatic ability and patients with decreased miR-211 have shorter median overall survival (OS) 48, 49. Lower miR-211 expression is positively associated with distant metastasis, lymph node metastasis, and poor prognosis in gastric cancer patients 32. The expression of miR-211 is reversely correlated with lymph node metastasis, distant recurrence, as well as clinical stage in patients with CRC 42. In HCC, patients with downregulated miR-211 have poor OS 15. Survival analysis indicated that low miR-211 expression is associated with shorter survival time in patients with OC 18, 37. In BC, low miR-211 expression is associated with poor TNM stage, lymph nodes metastasis and poor OS and miR211 serve as an independent biomarker for predicting overall survival of BC patients 35. High miR-211 level is associated with advancement of histological grade and MSTS stage in chondrosarcoma, and is identified as a predictor for poorer OS of chondrosarcoma patients 40. Besides, in TNBC, high plasma miR-211 levels are associated with brain metastasis and poor survival of patients. The research demonstrates that high plasma levels of miR-211 can be used as a predictor of brain metastasis and prognosis in TNBC 46.

## Biological role of miR-211 in cancer

### Proliferation and apoptosis

MiR-211 has been confirmed in a variety of cancers to regulate cell proliferation and apoptosis by targeting downstream targets (Table 2). Previous studies have shown that IL-10 promotes the cell proliferation and metastasis in melanoma 50. IL-10 promotes downstream signaling pathways through binding to the IL-10 receptor (IL-10R), which consists of two different chains (IL-10Rα and IL-10Rβ). In melanoma, miR-211 inhibits cell proliferation by targeting IL-10Rα 51. Upregulated miR-211 suppresses anchorage-independent colony formation in melanoma 24. MiR-211 is confirmed to inhibit cells proliferation in thyroid cancer 26 and cervical cancer 38 by regulating secreted protein acidic and rich in cysteine (SPARC), which is an extracellular matrix glycoprotein. SRY-related HMG box transcription factor 11 (SOX11), a member of the SOX transcription factors, promotes thyroid cancer cells proliferation and is downregulated by miR-211 25. It is reported that miR-211 inhibits the proliferation of triple-negative breast cancer cells (TNBC) 47. Sirtuin 1(SIRT1), which is a conserved NAD-dependent deacetylase, mediates deacetylation of p53 and regulates cell survival and apoptosis by this mechanism and thus potentially affects tumorigenesis 52. By targeting SIRT1, miR-211 reduces breast cancer cell viability and induces apoptosis 16. SRC Kinase Signaling Inhibitor 1 (SRCIN1) is identified to be regulator for affecting cell proliferation and migration in lung cancer 53. MiR-211 is indicated to regulate the expression of SRCIN1 in NSCLC, thus attenuating the proliferation of cancer cells 54. Upregulated miR-211-3p inhibits the proliferation of NSCLC cells by suppressing Zinc-figure protein 217 (ZNF217) 45. ZNF217 is known as a member of the Kruppel-like family of transcriptional factors and works as an important effector stimulating oncogenicity during multiple cancer processes 55. Ezrin is a membrane cytoskeleton cross-linker protein, and Ezrin phosphorylation regulates cell migration, mechanical properties, and cytoskeletal organization 56. Overexpressed miR-211 significantly decreases cell proliferation in tongue cancer via targeting Ezrin/Fak/Src signaling 57. MiR-211 inhibits HCC cells proliferation by downregulating special AT-rich sequence-binding protein-2 (SATB2), which is upregulated in HCC 27. ZEB2 acts as an oncogene to promote cells proliferation and inhibit cells apoptosis in HCC, and is regulated by miR-211^28^. Moreover, miR-211 suppresses HCC cell proliferation by targeting Acyl‑CoA Synthetase Long Chain Family Member 4 (ACSL4), which can promote the malignant phenotype in HCC 15. Overexpressed miR-211 suppresses HCC cell proliferation and angiogenesis by targeting Fork head box D1 (FOXD1) 29. FOXD1 dysfunction is linked to different pathologies and is known as one of the mediators for angiogenesis in various types of tumors 58. Some studies have indicated that the single-nucleotide polymorphism (SNP) is related to colon cancer occurrence and development 59. SNP of rs187960998 in miR-211 inhibits colon cancer cell proliferation by upregulating chromodomain-helicase-DNA binding protein 5 (CHD5), which acts as a tumor suppressive gene 60. SRY-related HMG box 4 (SOX4) is a member of the C subgroup of the SOX transcription factor family, and is a key transcription factor involved in a number of development procedures, including tumorigenesis 61. MiR-211 inhibits cells proliferation by reducing SOX4 in gastric cancer 31 and cervical cancer 30. MiR-211 may be a tumor suppressor in RCC to attenuate the proliferation and induce cell apoptosis of cancer cells 34. Upregulated miR-211 obviously suppresses cells proliferation and promotes cell apoptosis via downregulating histone deacetylase9 (HDAC9) in bladder cancer 35. HDAC9 as a target of miRNAs is involved in the progression of carcinogenesis through posttranscriptional regulation 62. MiR-211 is reported as a tumor suppressor to suppress proliferation and induce apoptosis via downregulating Cyclin D1 and CDK6 in OC 36. Overexpressed miR-211 apparently inhibits proliferation, xenograft growth, and induces apoptosis by suppressing PHD finger protein 19 (PHF19) in OC 37. PHF19 is a component of the polycomb group of proteins and negatively regulated by miR-211. Knockdown of miR-211 promotes the viability and proliferation of OC cells 63.

Contrary to the anti-proliferative effect reported above, emerging researches indicate miR-211 may promote cell proliferation various types of cancer. In details, MiR-211 facilitates the proliferation of osteosarcoma cells and inhibits cellular apoptosis 39. By the downregulation of Von Hippel-Lindau (VHL), miR-211 may function as an oncogenic in chondrosarcoma to modulate cell proliferation 40. MiR-211 may function as an oncogenic in NSCLC to enhance cell proliferation and colony formation by regulating SRCIN1 43. Furthermore, miR-211 promotes the cell proliferation through targeting EPH receptor B6 (EPHB6) in NSCLC 44. EPHB6, belongs to the Eph receptors family, that is found reduced in gastric carcinoma and correlated with malignancy indicators for cancer 64. In CRC, miR-211-3p also serves as an oncogene. The overexpressed miR-211-3p may facilitate CRC cell proliferation by targeting CDK6 65. CDK6 is recognized not only as a typical cyclin-dependent kinase but as a transcriptional regulator which regulates the transcription of a number of genes 66.

In OSCC, the roles for miR-211 as a tumor suppressor and as an oncomiR have been reported. In details, upregulated miR-211 increases the proliferation anchorage-independent colony formation of OSCC cells 14. In contrast to the above study, Guo *et al.* found that miR-211 suppresses cells proliferation in OSCC 67. These researches may suggest that the role of miR211 in cancers is related to tumor heterogeneity.

### Migration and invasion

Metastasis as main lethal feature of cancer is a multistep process, including tumor cell dissemination from the initial tumor and colonizing distant organs. The improved capacity of cancer cells to undergo migration and invasion contributes to the first step of this process. MiR-211 has been confirmed to regulate cells migration and invasion in various cancers.

**Figure 1:**
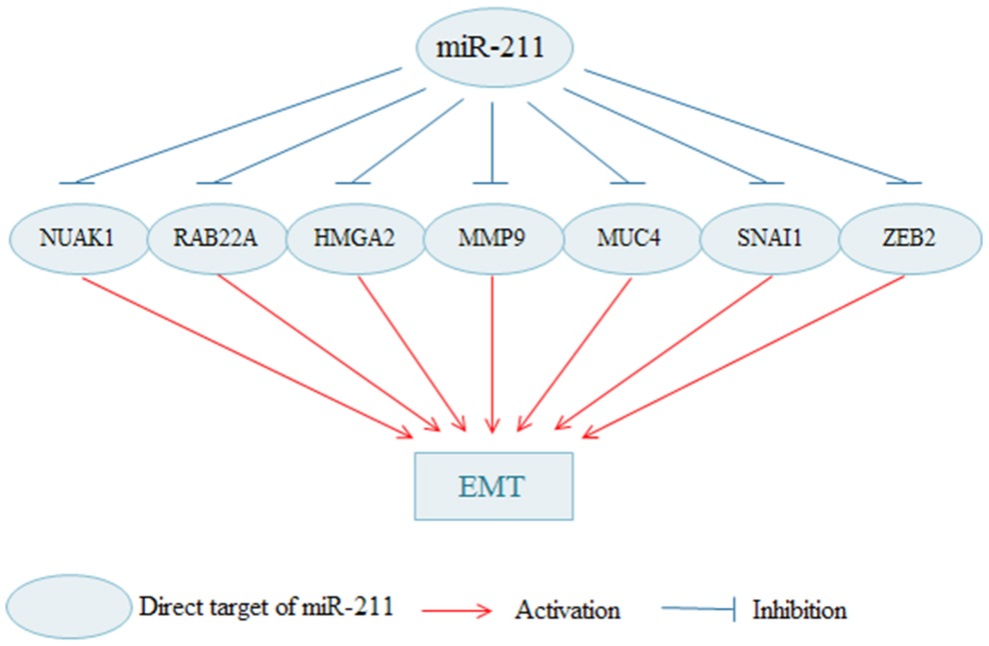
** The miR-211 regulates EMT in cancer cells.** By targeting various molecules, miR-211 inhabits the process of the EMT; thus, inhibiting the migration and invasion of cancer cells. EMT: epithelial-to-mesenchymal transition.

Cancer cells can acquire the migration and invasion capacity through the EMT progress. Various researches suggest that miR-211 is implicated to inhibit various genes that are involved in EMT (Fig. 1). MiR-211 contributes to cell adhesion and inhibits invasion by targeting NUAK Family SNF1-like Kinase 1 (NUAK1) in melanoma 68. NUAK1 (also named ARK5) is a member of AMPK catalytic subunit family involved in invasion and metastasis of malignant tumor 69. MiR-211 can inhibits EMT of melanoma cells via downregulating RAB22A, which is negatively regulated by MiR-211 22. HMGA2, a non-histone architectural transcription factor, influences a variety of biological processes, such as the cell cycle process, apoptosis, DNA damage repair process, senescence and EMT 70. MiR-211 suppresses breast cancer cells migration, invasion and EMT phenotype and through the downregulation of HMGA2 71. Matrix metalloproteinase 9 (MMP9) has been widely found to link to the pathology of cancers, such as invasion, metastasis and angiogenesis 72. MiR-211 suppresses cell invasion and cell EMT process by reducing MMP9 in gastric cancer 32. MiR-211 suppresses invasion and EMT of cervical cancer cells by targeting MUCIN-4(MUC4) 73. MUC4, a high molecular weight glycoprotein, is found to be associated with enhanced cancer cell invasion and EMT in several types of cancers 74. In addition, MiR-211-5p attenuates cell migration in HCC through targeting ZEB2 28. Snai1 family transcriptional repressor 1 (SNAI1) is zinc-finger transcription factor and is proved to induce EMT in cancer 75. Upregulated miR-211 inhibits cell migration and invasion by downregulating SNAI1 in RCC 33. Knockdown of miR-211 promotes the migration, invasion and EMT of OC cells 63.

**Figure 2:**
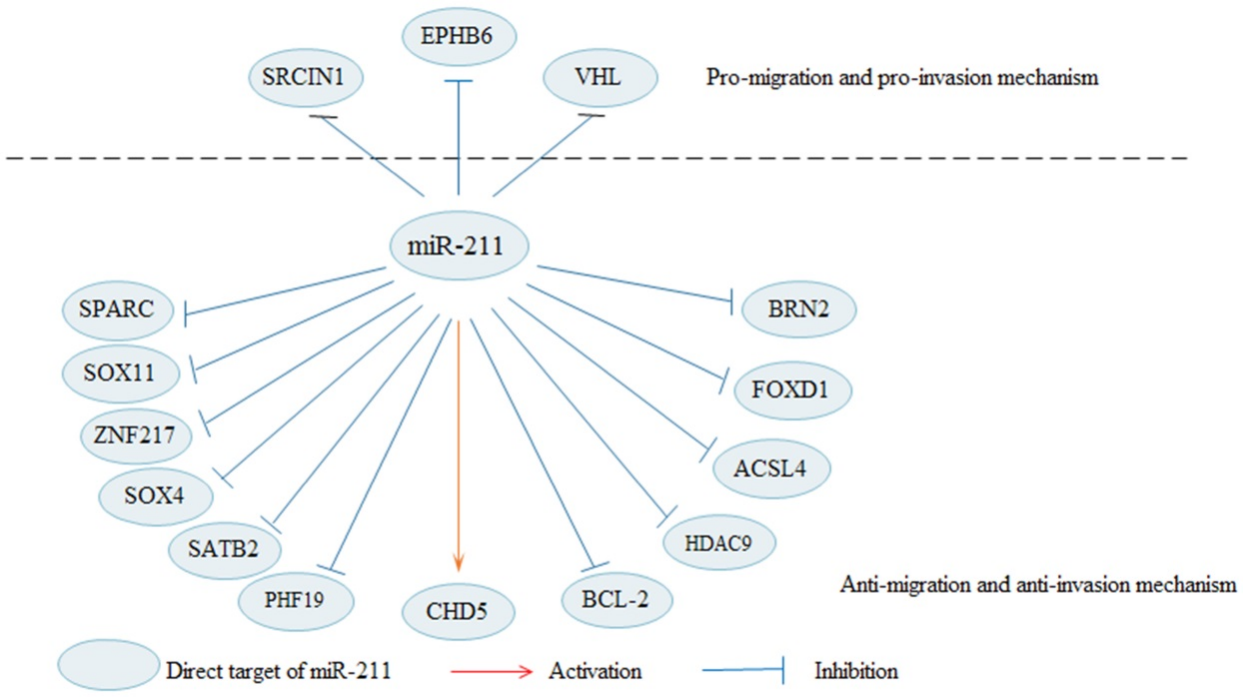
** Apart from the EMT, miR-211 regulates cell migration and invasion in cancers through other pathways.** By targeting BRN2, SPARC, SOX11, among others, miR-211 suppresses cell migration and invasion in cancers. In contrary, miR-211 promotes cell migration and invasion in cancers by targeting SRCIN1, EPHB6 and VHL.

In addition to the EMT process, miR-211 can also regulate the metastasis of cancer cells by influencing other pathways (Fig. 2). MiR-211 is indicated to decreases cells migration and invasion in malignant melanomas 20. Upregulated miR-211 decreases the invasive potential of melanoma cells through targeting BRN2, which is an important transcription factors 23, 76. In melanoma, Bcl-2 overexpression increases cell migration, and overexpression of miR-211 in Bcl-2 overexpressing cells can rescue cell migration 19. RAB22A is a member of the Rab family of small GTPases, and the oncogenic role of RAB22A is also observed in several types of cancer 77. In a xenograft model, miR-211 exhibits a dual role in melanoma progression, facilitating cell proliferation while suppressing metastatic spread 78. MiR-211 acts as a tumor suppressor in in thyroid cancer to modulate cells migration and invasion by decreasing SOX11 25. Besides, it is also reported that miR-211 can suppress cells invasion by regulating SPARC in thyroid cancer 26 and cervical cancer 38. In gastric cancer 31 and cervical cancer 30, SOX4 is identified a target of miR-211, and promotes cells invasion. In HCC, miR-211 suppresses cells invasion by targeting SATB2, which is negatively regulated by miR-211 27. In addition, miR-211 can inhibit HCC cells migration and invasion via downregulating ACSL4 15. Upregulated miR-211 inhibits HCC cells migration and angiogenesis through regulating FOXD1 29. Enforced expression of miR-211 significantly decreases cell invasion and migration in pancreatic cancer 49. MiR-211 may be a tumor suppressor in RCC to inhibit cells migration and invasion 34. Through regulating HDAC9, MiR-211 markedly suppresses bladder cancer cells migration and invasion 35. In OC, upregulated miR-211 apparently suppresses migration by inhibiting PHF19 37.

MiR-211 is confirmed as an oncogenic function in multiple cancers to regulate cells migration and invasion via targeting different genes. Upregulated miR-211 increases the migration of OSCC cells 14. MiR-211 facilitates cell migration in osteosarcoma 39. Furthermore, miR-211 may accelerate cell migration by the down-regulation of VHL in chondrosarcoma 40.

In some tumors, miR-211 can act as both a tumor suppressor and a tumor promoter. In detail, SNP of rs187960998 in miR-211 inhibits colon cancer cell invasion by upregulating CHD5 60. Another research shows that miR-211-3p acts as a tumor promoter to increase CRC cells invasion 65. In NSCLC, overexpressed miR-211-3p inhibits cells migration by downregulating ZNF217 45. However, miR-211 may also function as an oncogenic to facilitate cell migration and invasion by regulating SRCIN1 43 or EPHB6 44 in NSCLC. These results suggest that the effects of miR-211 may be strongly related to tumor heterogeneity. Furthermore, miR-211 inhibits cells migration and invasion ability, and reverse the EMT phenotype by downregulating high mobility group A2 (HMGA2) in breast cancer 71. MiR-211 suppresses cell invasion and migration in triple-negative breast cancer 47. However, high miR-211 facilitates brain metastasis by increasing the cells trans-blood-brain barrier (BBB) migration ability, BBB adherence, and stem cell properties in TNBC. SOX11 is the downstream target of miR-211 and negatively regulated by miR-211 46. Interestingly, miR-211 studies on breast cancer shows that miR-211 targets different genes to inhibit breast cancer proliferation, invasion and migration, but promotes metastasis. These results may indicate that miR-211 may play a different role in different stages of breast cancer progression.

### Chemoresistance

**Figure 3:**
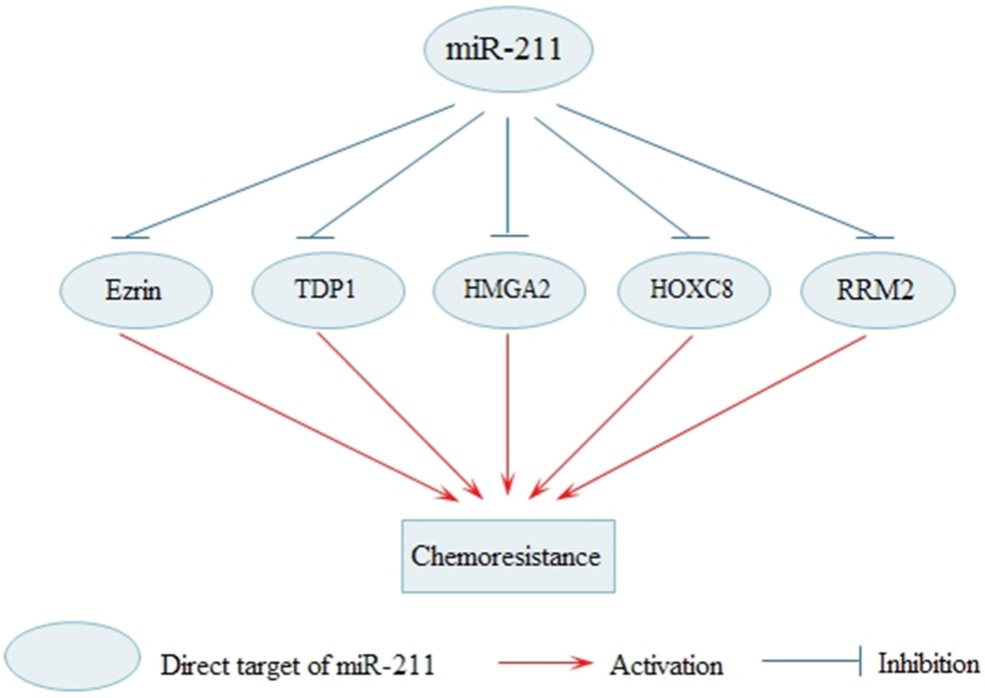
** MiR-211 regulates the sensitivity of cancer cells to the chemotherapy.** MiR-211 enhances the sensitivity of cancer cells to the chemotherapy through the inhibition of Ezrin, TDP1, HMGA2, HOXC8 and RRM2.

Systemic chemotherapy is important treatment for cancer but long-term administration of chemotherapy may lead to the development of chemoresistance. Effective therapies to reverse chemoresistance in cancer are still in exploration. At present, effective treatments to reverse cancer chemoresistance are still being explored. Various researches indicate that dysregulated miR-211 is involved in the molecular mechanisms of chemoresistance (Fig. 3). Overexpression miR-211 significantly decreases cell cisplatin resistance in tongue cancer via targeting Ezrin/Fak/Src signaling 57. The upregulated miR-211 enhances chemosensitivity of breast cancer cells by downregulating HMGA2 71. In addition, upregulated miR-211 in PDAC increased the sensitivity to gemcitabine by reducing ribonucleotide reductase subunit 2(RRM2) 49. Elevated ribonucleotide reductase (RR) activity and over expression of RRM2 pronouncedly increases the drug-resistant properties of human malignant cells 79. MiR-211 enhances platinum chemosensitivity through inhibiting DNA damage response (DDR) in ovarian cancer, and TDP1, one of the DDR genes, is identified as a target of miR-211 18. Multiple studies have shown that the enforced DDR is one of the most pivotal mechanisms for resistance to platinum-based chemotherapy 80. Moreover, miR-211 can promote cell sensitivity to paclitaxel (PTX) by targeting HOXC8 81. Besides, the finding also shows that miR-211 contributes to BRAF inhibitor resistance in melanoma 41. The BRAF V600E mutation which is common in melanoma, is the focus of recently developed BRAF inhibitors (BRAFi), such as vemurafenib and dabrafenib 82.

### Cell cycle

**Figure 4:**
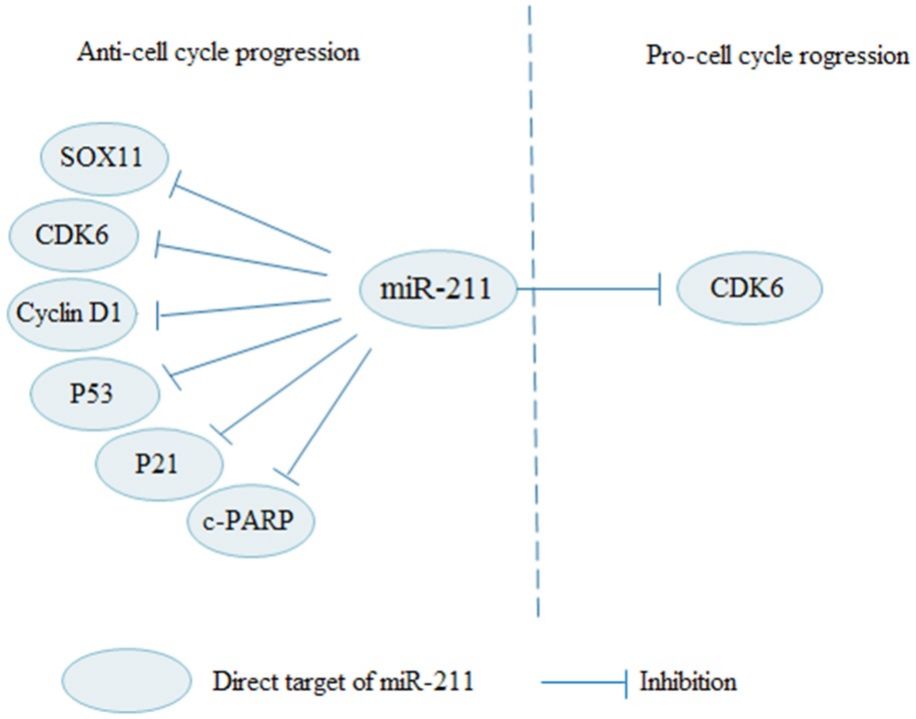
MiR-211 regulates cell cycle progression in cancers via various signaling pathways. MiR-211 suppresses cell cycle progression through regulating SOX11, CDK6, Cyclin D1, P53, P21 and c-PARP. In contrary, miR-211 promotes cell cycle progression by inhibiting CDK6.

Numerous signaling pathways and checkpoints are involved in regulating the cell cycle. Studies on cell cycle regulation provide a new direction for cancer treatments. A large number of studies have shown that miR-211 regulates cell cycle by regulating multiple targets (Fig. 4). Various factors affect the G1-S phase transition, including cyclin D1, cyclin-dependent kinases (CDKs), and CDK inhibitors (CKIs). In OSCC, miR-211 can increase the number of cells in G1 phase, and p53, p21, c-PARP levels, and decrease cyclin D1 levels 67. Overexpressed miR-211 induces cell cycle arrest and apoptosis by targeting SOX11 in thyroid tumor 25. As a tumor suppressor, miR-211 inhibits cell proliferation and induces cell apoptosis by down-regulating cyclin D1 and CDK6 in OC to block cells in G0/G1 phase 36. Apart from its inhibitory effect, miR-211 also acts as a facilitator in cell cycle process. MiR-211-3p promotes CRC cell proliferation and decreases cell cycle arrested at G1 phase by targeting CDK6 65. Therefore, research on miR-211 can better reveal the molecular mechanism of miR-211 in cell cycle regulation.

### Cancer stem cell

Cancer stem cells (CSCs) are a small group of solid tumor cells with self-renewal, differentiation properties and tumorigenic potential 83. CSCs are thought to be closely related to tumor formation, metastasis and recurrence 84. Metastatic cancer stem cells (MCSCs) are a subpopulation of cancer cells with stem cell properties, invasion capabilities, and trafficking functions 85. MiR-211 levels are found to be regulating the stemness properties of CSCs. High expression of miR-211 enhances stemness properties of cancer cells to promote brain metastasis in TNBC by regulating the SOX11/NGN2-dependent axis 46.

### The targets of miR-211

#### SOX transcription factor family

SRY-related HMG box (SOX) transcription factors have been recognized to play a critical role in a variety of tumor biological processes, including tumorigenesis, proliferation and metastasis, and change in the tumor microenvironment 86. Among the more than 20 members of the SOX family, SOX11 and SOX4 have been shown to play important roles as targets of mir211 in a variety of cancers.

SOX11 is a crucial member of the SOX transcription factors, which encodes a neural transcription factor, have functional role in multiple cancers 87. SOX11 exerts tumor-stimulative effects in various tumors by promoting cell survival and proliferation, and initiating metastasis angiogenesis 88. In a limited number of malignant tumors, SOX11 acts as a tumor suppressor by inhibiting cell proliferation and metastasis, as well as suppressing the maintenance of cancer-initiating cells 89. MiR-211 acts as a tumor suppressor or tumor promoter in malignancies by targeting SOX11. In detail, miR-211 modulates cells proliferation, migration and invasion by decreasing SOX11 in thyroid cancer 25. High miR-211 increases tumor cell adhesion, trans-blood-brain barrier (BBB) ability and cancer stem cell capability to promote brain metastasis in TNBC. SOX11 is the downstream targeting molecules of miR-211 and negatively regulated by miR-211 46. These results suggest that the role of miR-211 in tumors is closely related to the function of its targets.

SOX4 is thought to be involved in the development of many diseases, including malignancies 90. Numerous findings highlight the explicit role of SOX4 in regulating cancer progression by affecting the ability of cancer cells to survive, migrate and invade 91. Emerging researches show that miR-211 targets SOX4 to regulate cancer progression. MiR-211 serves as a tumor suppressor to inhibit cells proliferation and invasion by reducing SOX4 in gastric cancer and cervical cancer 30, 31.

#### SPARC

SPARC is an extracellular matrix glycoprotein secreted by kinds of cells and has been shown to be associated with the cancer progression 92. Numerous studies have confirmed that SPARC affects cancer progression by modulating cell adhesion, invasion, metastasis, and angiogenesis 93. However, there are still emerging studies highlighting the role of SPARC in cancer cells proliferation regulation, such as oral squamous cell carcinoma 94, liver cancer 95, and cervical cancer 96. SPARC is involved in regulating multiple cancers progression as a target gene of miR-211. In thyroid cancer, SPARC acts as an oncogene to promote cells proliferation and invasion, and is regulated by miR-211 26. Besides, miR-211 inhibits cervical cancer cells proliferation, invasion and migration by inhibiting SPARC 38.

#### SNAI1

SNAI1 is a zinc finger transcription factor and functions as a driver of cancer progression 97. Numerous studies have shown that Snail1 is a crucial inducer of EMT and plays a vital role in tumor metastasis 98. Recently, the relationship between SNAI1 and miRNAs is reported in various cancers 99. Moreover, Wang K *et al.* 33 suggests that miR-211 suppresses the cells migration and invasion by the downregulation of SNAI1 in RCC, emphasizing the significance of the miR211/SNAI1 axis in cancer.

#### ZEB2

ZEB2, also known as smad-interacting protein 1 (SIP1), belongs to the zinc-finger E-box binding protein (ZEB) family and has been shown to induce cancer progression 100. Existing studies indicate that ZEB2 promotes cancer metastasis by inducing EMT 101. ZEB2 is modulated by multiple miRNAs in cancers, such as miR-139-5P 102 and miR-338-3p 103. Besides, miR-211 suppresses the cells survival and attenuates the cells metastasis through downregulating ZEB2 in HCC 28.

#### The other targets of miR-211

In addition to the above targets, miR-211 also acts on a range of other targets. The targets and the effects of miR-211 are explicitly shown in Table 3. Overall, miR-211 plays an important role in cancer progression in diverse types of cancer, through regulating different targets.

## Factors regulating the expression of miR-211 in cancer

### LncRNA and CircRNA

Emerging researches indicate that miRNAs, long non-coding RNAs (lncRNAs), and circular RNAs (circRNAs), which are major members of non-coding RNAs (ncRNAs), are involved in cancer development. Competing endogenous RNAs (ceRNAs) networks describe competitive binding between sponge RNAs and miRNA targets as well as the regulation of miRNA targets activity 104. Various lncRNAs and circRNAs are indicated to participate in the regulation of miR-211 in different cancers (Table 4).

LncRNA KCNQ1OT1 facilitates proliferation and chemo-resistance in tongue cancer by acting as a sponge of miR-211 through Ezrin/Fak/Src signaling 57. LncRNA MCM3AP-AS1 promotes the papillary thyroid cancer cell proliferation and invasion by regulating miR-211/SPARC axis 26. In breast cancer, lncRNA NEAT1 is upregulated. NEAT1 negatively regulates miR-211 expression via acting as an endogenous sponge 71. In breast cancer, LncRNA SNHG15 facilitates cells proliferation and migration through sponging miR-211-3p 17. In addition, LncRNA TTN-AS1 is significantly upregulated and regulates the proliferation and invasive and migratory abilities of triple-negative breast cancer cells by inhibiting miR-211 47. Upregulated LncSNHG15 accelerates the NSCLC cells proliferation and migration by sponging miR-211-3p 45. Moreover, LncRNA DGCR5 suppresses cell proliferation, migration, and invasion through modulating miR-211/EPHB6 axis in NSCLC 44. Studies show that decreased microphthalmia-associated transcription factor (MITF) are associated with increased proliferation. MITF/miR-211 axis inhibits the invasive program in melanoma by blocking adhesion 105. CD44, a non-kinase transmembrane glycoprotein, is frequently shows alternative spliced variants that are considered to play a significant role in cancer development and progression 106. LncRNA-uc002kmd.1 expression is highly expressed in CRC. The results indicate that lncRNA-uc002kmd.1 may upregulate CD44 by competing for miR-211-3p, subsequently regulating cell migration and proliferation in CRC 107. LncRNA TUSC7 inhibits proliferation and invasion of CRC cells and induces cell cycle arrest through completely sponging miR-211-3p. TUSC7 is negatively correlated with miR-211-3p and CDK6 is a downstream target of miR-211-3p 75. Likewise, another research also indicates that LncRNA TUSC7 suppresses the proliferation, migration of osteosarcoma cells and induces cellular apoptosis, which is mediated by miR-211. miR-211 is inhibited remarkably by TUSC7 and the reciprocal negative regulation exists between TUSC7 and miR-211^39^. In HCC, LncRNA NORAD facilitates cells proliferation, migration and vessel formation abilities by sponging miR-211 29. LncRNA MALAT1 can sponge miR-211 as a competing endogenous RNA thus facilitating the OC progression 37.

CircCCDC66 acts as a sponge of miR-211 and promotes NSCLC cell proliferation by regulating miR-211/SRCIN1 axis 54. Besides, irc_0008285 is remarkably elevated in cervical cancer and facilitates cervical cancer cells proliferation and invasion through regulating miR-211-5p/SOX4 Axis 30. Homeobox C8 (HOXC8) is remarkably overexpressed in cancer and identified as a regulator in resistance of hepatocellular carcinoma to oxaliplatin 108. Furthermore, circNRIP1 promotes the resistance of OC cells to paclitaxel through affecting the miR-211-5p/HOXC8 axis 81.

### Other factors

Ligustrazine (LSZ) can significantly increase the expression of miR-211 in OC. Ligustrazine inhibits the OC cells proliferation and migration by regulating miR-211 63. Alpinetin suppresses OSCC cells proliferation by upregulating miR-211 and Notch Pathway deactivation 67. Adipocytes secretes the cytokines IL-6 and TNF-α, which induces a proliferative-to-invasive phenotypic switch in melanoma by decreasing the expression of miR-211 78. Protein kinase RNA (PKR)-like ER kinase (PERK), which is an ER transmembrane protein kinase, catalyzes serine 51 phosphorylation on eIF2α, thus, resulting in decreased protein synthesis 109. A PERK-miR-211 axis inhibits circadian regulators and protein synthesis to facilitate cancer cell survival in Burkitt's lymphoma, and miR-211 diametrically modulates Bmal1through distinct mechanisms 110.

## Conclusion

Emerging experimental evidence reveal that miR-211 play a crucial role in cancer, because it is involved in various biological processes, including cell proliferation, apoptosis, invasion, and metastasis. The complicated regulatory network targeting miR-211 and its target genes remain incompletely understood. Nevertheless, miR-211 is a potential prognostic biomarker, even if its extensive functions and dysregulation are associated with multiple types of cancer.

More attractive is miR-211-based therapy that replaces miR-211 in cancer cells and regains control of numerous signaling pathways regulated by endogenous miR-211. Also, overexpressed miR-211 can enhance the sensitivity of cancer cells to chemotherapy, indicating a new approach for overwhelming drug resistance. The implementation of specific miRNA mimics has been proved to have strong tumor inhibitory ability in experimental tumor models, such as miR-34 111.

However, miRNA-based therapy still has some limitations, for instance, the lack of dosage requirements and its administration. The premature termination of the two registered clinical trials using the miR-Rx34 were due to serious immune-related adverse events that may be caused by off-target effects due to high doses of miR-34 mimic 112. For all that, work is under way to address these limitations, and recent advances in drug-delivered systems provide a technical solution for the administration and delivery of miRNA mimics. These discoveries will usher in a new era of RNA-based therapy, especially in the field of cancer treatment. Thence, there are two main goals that must be realized in order to enter the RNA therapeutic era: further improve the drug delivery strategy and determine the possible adverse reactions of these molecules.

In summary, further researches should be implemented before miR-211 can be widely used as a prognostic biomarker and therapeutic target, but its dysregulation in numerous cancers, and participation in a variety of biological functions, makes it a promising molecule target for cancer therapy.

## Figures and Tables

**Table 1:** Expression patterns and prognostic value of miR-211 in various types of cancer

System	Type	Expression	Prognostic value	Reference
Respiratory system	NSCLC	Downregulation	Higher lymph node metastasis	45
		Upregulation	/	43,44
Skeletal system	Osteosarcoma	Upregulation	/	39
	Chondrosarcoma	Upregulation	Higher Histological stage, poorer overall survival (OS)	40
Digestive system	OSCC	Upregulation	The most advanced nodal metastasis, vascular invasion, and poor prognosis	14
	Gastric cancer	Downregulation	Distant metastasis, lymph node metastasis and poor outcome	31,32
	HCC	Downregulation	Poor OS	15,27-30
	Pancreatic cancer	Downregulation	Shorter median OS and higher metastatic ability	48,49
	CRC	Downregulation	Lymph node metastasis, distant metastasis, and poor cancer stage	42
Urinary system	RCC	Downregulation	/	33,34
	Bladder cancer	Downregulation	Poor TNM stage, lymph nodes metastasis and poor OS	35
Reproductive system	Ovarian cancer	Downregulation	Shorter OS	18, 36,37
	Cervical cancer	Downregulation	/	38
Others	Breast cancer	Downregulation	Poor TNM stage, lymph nodes metastasis and poor OS	17,47
		Upregulation	brain metastasis and poor survival	46
	Thyroid cancer	Downregulation	/	25,26
	Melanoma	Downregulation	/	20-24
		Upregulation	/	41

NSCLC: non-small cell lung cancer; OSCC: oral squamous cell carcinoma; CRC: colorectal cancer; HCC: hepatocellular carcinoma; RCC: renal cell carcinoma.

**Table 2:** MiR-211 regulates cell proliferation and apoptosis in multiple cancers

Cancer type	Downstream target	Effect	Reference
Thyroid cancer	SPARC	Proliferation inhibition	26
	SOX11	Proliferation inhibition	25
NSCLC	SRCIN1	Proliferation inhibition	54
	ZNF217	Proliferation inhibition	45
	EPHB6	Promotes proliferation	44
	SRCIN1	Promotes proliferation	43
Tongue cancer	Ezrin	Proliferation inhibition	57
Gastric cancer	SOX4	Proliferation inhibition	30
HCC	SATB2	Proliferation inhibition	27
	ZEB2	Proliferation inhibition and Apoptosis induction.	28
	ACSL4	Proliferation inhibition	15
	FOXD1	Proliferation inhibition	29
CRC	CHD5	Proliferation inhibition	60
	CDK6	Promotes proliferation	65
Bladder cancer	HDAC9	Proliferation inhibition and Apoptosis induction	35
Ovarian cancer	Cyclin D1/CDK6	Proliferation inhibition and Apoptosis induction	36
	PHF19	Proliferation inhibition and Apoptosis induction	37
Cervical Cancer	SPARC	Proliferation inhibition	38
	SOX4	Proliferation inhibition	31
Melanoma	IL-10Rα	Proliferation inhibition	51
Chondrosarcoma	VHL	Promotes proliferation	40
Breast cancer	SIRT1	Proliferation inhibition and Apoptosis induction	16

NSCLC: non-small cell lung cancer; HCC: hepatocellular carcinoma; RCC: renal cell carcinoma.

**Table 3:** Targets of miR-211 known to be involved in cancers

Target	Cancer type	Effect	Reference
SOX11	Thyroid cancer	Proliferation, migration, and invasion	25
SOX11	Breast cancer	Metastasis	46
HMGA2	Breast cancer	Migration and invasion and EMT	71
SIRT1	Breast cancer	Proliferation and apoptosis	16
SPARC	Thyroid cancer	Proliferation and invasion	26
SPARC	Cervical cancer	Proliferation, invasion and migration	38
MMP9	Gastric cancer	Invasion and EMT	32
SOX4	Gastric cancer	Proliferation and invasion	30
SOX4	Cervical Cancer	Proliferation and invasion	31
MUC4	cervical cancer	Invasion and EMT	72
SRCIN1	NSCLC	Proliferation, migration and invasion	43
SRCIN1	NSCLC	Proliferation	54
ZNF217	NSCLC	Proliferation and migration	45
EPHB6	NSCLC	Proliferation, migration and invasion	44
Ezrin	Tongue cancer	Proliferation	57
SATB2	HCC	Proliferation and invasion	27
ZEB2	HCC	Proliferation, apoptosis and metastasis	28
ACSL4	HCC	Proliferation, migration and invasion	15
FOXD1	HCC	Proliferation and migration	29
SNAI1	RCC	Migration and invasion	33
CHD5	CRC	Proliferation inhibition invasion	60
CDK6	CRC	Promotes proliferation	65
HDAC9	Bladder cancer	Proliferation, apoptosis, migration and invasion	35
Cyclin D1/CDK6	Ovarian cancer	Proliferation and apoptosis	36
PHF19	Ovarian cancer	Proliferation, apoptosis and migration	37
RAB22A	Melanoma	EMT	22
IL-10Rα	Melanoma	Proliferation	51
NUAK1	Melanoma	Adhesion and invasion	68
BRN2	Melanoma	Migration and invasion	23
Bcl-2	Melanoma	Migration and invasion	19
VHL	Chondrosarcoma	Proliferation and migration	40

NSCLC: non-small cell lung cancer; CRC: colorectal cancer; HCC: hepatocellular carcinoma.

**Table 4:** Regulatory factors regulate the expression of miR-211 in various cancers

Type of Factors	Factor	Cancer type	Reference
LncRNA	lncRNA KCNQ1OT1	Tongue cancer	57
	LncRNA MCM3AP-AS1	Thyroid cancer	26
	lncRNA NEAT1	Breast cancer	71
	LncRNA SNHG15	Breast cancer	17
	LncRNA TTN-AS1	Breast cancer	47
	LncSNHG15	NSCLC	45
	LncRNA DGCR5	NSCLC	44
	LncRNA-uc002kmd.1	CRC	107
	LncRNA TUSC7	CRC	75
	LncRNA TUSC7	Osteosarcoma	39
	LncRNA NORAD	HCC	29
	LncRNA MALAT1	Ovarian cancer	37
CircRNA	CircCCDC66	NSCLC	54
	Circ_0008285	Cervical Cancer	30
	CircNRIP1	Ovarian cancer	81
Others	Ligustrazine	Ovarian cancer	63
	Alpinetin	OSCC	67
	Adipocytes	Melanoma	78
	PERK	Burkitt's lymphoma	110

NSCLC: non-small cell lung cancer; OSCC: oral squamous cell carcinoma; CRC: colorectal cancer; HCC: hepatocellular carcinoma.
